# Why central venous pressure falls below supine levels in weightlessness

**DOI:** 10.1152/japplphysiol.00271.2022

**Published:** 2022-06-16

**Authors:** Jay C. Buckey, Mimi Lan

**Affiliations:** ^1^Department of Medicine, Geisel School of Medicine at Dartmouth, Lebanon, New Hampshire; ^2^Thayer School of Engineering at Dartmouth, Hanover, New Hampshire

**Keywords:** central venous pressure, gravitation, intrathoracic pressure, microgravity, numerical model, weightlessness

Central venous pressure (CVP) falls below supine levels upon entering weightlessness, but the reason for this has been debated. A commonly presented explanation for this reduction is that intrathoracic pressure falls dramatically in weightlessness (1, 2). In this theory, the pressure drop in the thorax is transmitted to the intrathoracic vasculature creating a drop in CVP. This explanation is based on comparing esophageal pressure measurements taken via esophageal balloon in the supine position on Earth to balloon measurements taken in weightlessness on parabolic flights (1). Esophageal pressure is commonly used as a surrogate for intrathoracic pressure and is measured using a balloon in the lower one-third of the esophagus, just posterior to the heart (3).

This explanation has problems. Although upright esophageal balloon measurements may reflect intrathoracic pressure accurately, supine measurements do not (3–5). In the supine position, mediastinal weight is known to elevate esophageal pressure beyond that of intrapleural pressure (6, 7). The heart and other tissues lying in front of the esophagus compress the balloon, increasing balloon pressure beyond intrathoracic pressure (Fig. 1*A*). This increase is supported by studies investigating postural effects on esophageal measurements, which show higher esophageal balloon pressures supine compared with upright, even though direct measurements of intrathoracic pressure do not show major differences between these postures (5). In weightlessness, without the force of gravity, tissue pressures are removed from the balloon, causing balloon pressure to drop. This drop, however, is not necessarily due to a major reduction in intrathoracic pressure but is instead due to the removal of gravitationally induced forces from the balloon. If this pressure change is ascribed primarily to changes in intrathoracic pressure this can lead to a misinterpretation of changes in intrathoracic pressure in weightlessness.

**Figure 1.**
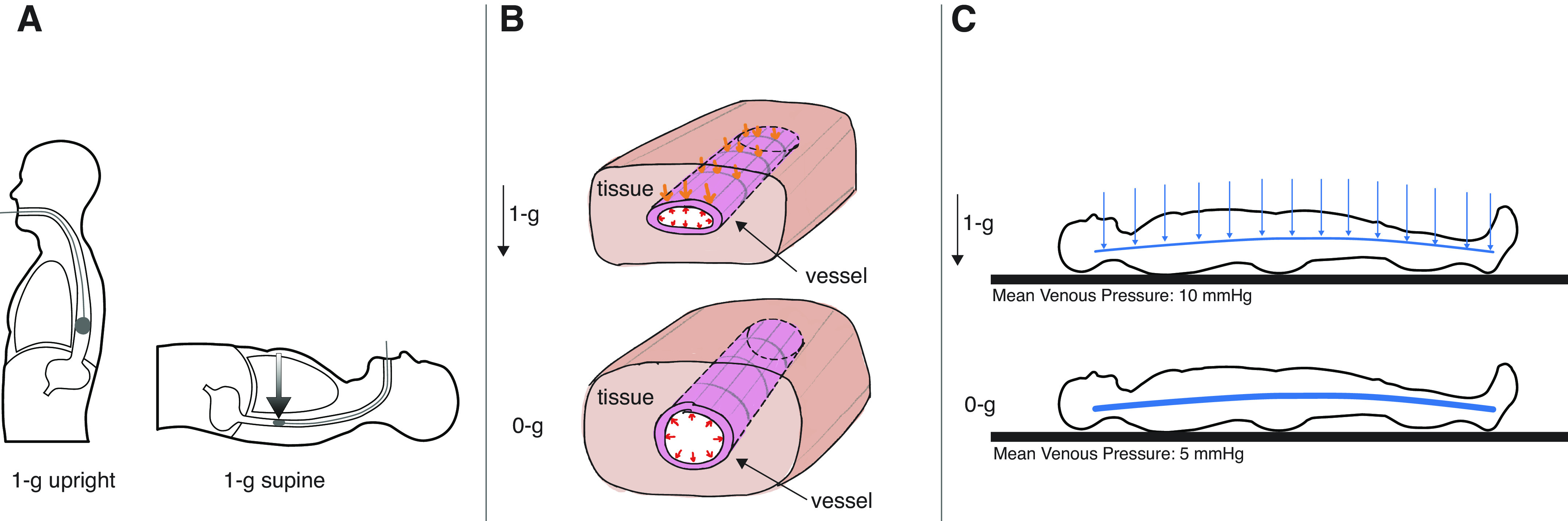
*A*: in the upright position the esophageal balloon measures intrathoracic pressure accurately. In the supine position, however, the weight of tissues above the balloon increase the measured pressure beyond intrathoracic pressure. *B*: schematic diagram of a proposed effect of removing tissue weight on the compliance of a vessel. *C*: schematic of the proposed changes in venous pressures when moving from the supine position to weightlessness. The removal of tissue weight allows the same amount of blood to be contained at a lower pressure. [Panel *C* adapted from Lan et al. (8) with permission from Elsevier Science and Technology Journals. Copyright © 2022 International Academy of Astronautics.]

In the seminal study on weightlessness and intrathoracic pressure by Videbaek and colleagues (2), esophageal pressure was measured both supine and upright before parabolic flight and then again during short-duration weightlessness exposure. The supine esophageal balloon pressure (1.5 mmHg) was compared with the weightless esophageal pressure (−4.1 mmHg) leading to the conclusion that intrathoracic pressure was reduced dramatically in weightlessness (a 5.6 mmHg reduction). This reduction in intrathoracic pressure was postulated to contribute to the reduced CVP seen in weightlessness and to increase the transmural pressure across the heart leading to increases in atrial and ventricular dimensions.

The problem with this conclusion, however, is that no allowance was made for the weight of the tissues that cause supine esophageal pressure to differ from intrathoracic pressure. Some authors suggest reducing measured supine balloon pressure by 3.7 mmHg to compensate (3, 9). When this is done, the reduction in intrathoracic pressure when entering weightlessness is much more modest (a 1.9 mmHg rather than a 5.6 mmHg reduction). This degree of change fits better with measurements of lung volume in weightlessness. If intrathoracic pressure were to be reduced dramatically, this should also lead to a substantial increase in lung volume, which is not seen (10). If upright esophageal balloon pressures are compared with microgravity this shows a slight increase rather than a decrease in intrathoracic pressure. Overall, the evidence for a major reduction in intrathoracic pressure in space is not strong.

An alternative explanation for the reduced CVP in weightlessness is that the removal of gravitational forces removes both hydrostatic forces within vessels and the pressures produced by the weight of tissues on the outside of blood vessels (4). Tissue weight produces compressive forces that act on the walls of veins and venules (11; Fig. 1*B*). Removing compressive forces from the walls of arteries, veins, and venules would increase vascular capacity and increase unstressed volume (i.e., the volume of blood contained at zero distending pressure). This reduces mean circulatory filling pressure, which affects venous pressures throughout the body. The increased compliance of the venous system allows venous blood to be contained in the same vascular space at a lower pressure (Fig. 1*C*). This overall reduction in venous pressure explains why not just CVP but also peripheral venous pressure is reduced in weightlessness (12). The converse also seems to be true. Increasing compressive forces with Gx centrifugation increases right atrial pressure (13).

Obesity leads to increased tissue pressures and so may also provide a way to understand the spaceflight results. De Divitiss et al. (14) performed right and left heart catheterization on 10 nonhypertensive subjects with obesity. Right atrial pressure was correlated positively with both weight and the degree of overweight. Similarly, Agarwal et al. (15) compared 10 surgical patients with overweight/obesity (91.8 ± 18 kg) with 10 normal weight surgical patients. The patients with obesity had right atrial pressures 120% higher than the patients with normal weight. Alaud-din et al. (16) studied 30 patients with class III obesity. Pulmonary capillary wedge pressure was positively correlated with weight. This group remeasured 12 of their patients after they lost an average of 55 kg and showed that filling pressures had decreased. Obesity affects many body systems, and patients with obesity who lose weight may show changes in sodium handling, so its possible effects from weight loss other than changes in tissue weight affected venous pressures in the chest. Even so, the obesity results are compatible with the notion that increased tissue weight increases venous pressures at the heart.

Numerical modeling also offers a way to estimate the effects of removing tissue compressive forces. Removing those forces is not possible on Earth but can be simulated. We developed a multicompartment, lumped-parameter, numerical circulatory model that incorporates tissue-weight generated compressive forces exerted on vessels, as well as venous and arterial hydrostatic gradients (17). The model has been implemented using MATLAB Simscape Fluids (MathWorks, Natick, MA; 18) and shows that CVP falls in weightlessness relative to supine (19).

In sum, available evidence does not support changes in intrathoracic pressure as the primary mechanism for decreased CVP in space below supine values, although it may contribute. The more likely cause is that the removal of hydrostatic gradients and tissue compressive forces throughout the body leads to a reduction in mean circulatory filling pressure, which is reflected in reductions of both central and peripheral venous pressures. The increased stroke volume and cardiac output that occur despite the lower venous pressures are likely due to changes in cardiac compliance also produced by the removal of gravity (4). It is, however, very challenging to collect physiological measurements that would support this hypothesis directly.

Nevertheless, this hypothesis has implications for understanding some of the unique effects of weightlessness. The spaceflight associated neuro-ocular syndrome, for example, is sometimes thought to result from increased venous pressures in the head. If weightlessness is leading to a generalized reduction in venous pressures throughout the body, it is hard to postulate how head veins could sustain locally increased venous pressures. Instead, theories to explain SANS that take the likely reduction in venous pressures into account are needed (20).

## GRANTS

The development of the numerical models was supported by Grant CA03401 from the National Space Biomedical Research Institute through NCC 9–58 and by NASA EPSCoR Cooperative Agreement NNX13AD35A.

## DISCLOSURES

No conflicts of interest, financial or otherwise, are declared by the authors.

## AUTHOR CONTRIBUTIONS

J.C.B. conceived and designed research; J.C.B. and M.L. analyzed data; J.C.B. and M.L. interpreted results of experiments; J.C.B. and M.L. prepared figures; J.C.B. drafted manuscript; J.C.B. and M.L. edited and revised manuscript; J.C.B. and M.L. approved final version of manuscript.
